# TUG1 and H19 lncRNAs Can Predict Anti‐TNF Unresponsiveness in Patients With Ulcerative Colitis: A Machine Learning–Based Approach

**DOI:** 10.1155/mi/9078048

**Published:** 2026-02-28

**Authors:** Raheleh Heydari, Mohammad Javad Tavassolifar, Mohammad Hossein Derakhshan Nazari, Romina Roshannia, Shabnam Shahrokh, Maryam Parvizi, Mohammad Tayefeh Norooz, Anna Meyfour

**Affiliations:** ^1^ Basic and Molecular Epidemiology of Gastrointestinal Disorders Research Center, Research Institute for Gastroenterology and Liver Diseases, Shahid Beheshti University of Medical Sciences, Tehran, Iran, sbmu.ac.ir; ^2^ Research Center for Gastroenterology and Liver Diseases, Research Institute for Gastroenterology and Liver Diseases, Shahid Beheshti University of Medical Sciences, Tehran, Iran, sbmu.ac.ir; ^3^ Department of Pathology, School of Medicine, Shahid Beheshti University of Medical Sciences, Tehran, Iran, sbmu.ac.ir; ^4^ General Surgery Department, Modarres Hospital, Shahid Beheshti University of Medical Sciences, Tehran, Iran, sbmu.ac.ir

**Keywords:** anti-TNF-α therapy, H19, long noncoding RNA, TUG1, ulcerative colitis

## Abstract

The present study aimed to explore the association of long noncoding RNAs (lncRNAs) with inflammation, disease activity, and predicting response to anti‐tumor necrosis factor (TNF)‐α therapy in patients with ulcerative colitis (UC). Whole blood samples and inflamed biopsies were collected from 42 UC patients at baseline (W0) and week 14 (W14) after receiving anti‐TNF treatment as a discovery cohort. Colonoscopy images, histopathological, and clinical symptoms were used to monitor disease activity and response to treatment. LncRNA expression analysis showed increased expression of H19 in the active lesions of UC nonresponders (UCNs) compared to UC responders (UCRs) at baseline, whereas taurine upregulated gene 1 (TUG1) expression was lower. Higher expression of H19 in UCN compared to UCR was still observed at W14, whereas its expression was downregulated in UCR in the remission phase at W14 versus W0, suggesting it as a marker for monitoring disease activity. Moreover, colonic expression of H19 was positively correlated with erythrocyte sedimentation rate (ESR) and C‐reactive protein (CRP). In blood, both H19 and TUG1 showed increased expression in UCN versus UCR at baseline and W14. Three‐fold cross‐validation–based machine learning approach and receiver operating characteristic (ROC) curve analysis showed that H19 and TUG1 had strong predictive performance for anti‐TNF response, with accuracies of 90% and 93% in tissue and 85% and 60% in blood, respectively. In the validation cohort (12 UCR and 10 UCN), expression patterns were reproduced, and predictive performance remained high. H19 showed better accuracy in blood (85%) than tissue (70%), while TUG1 performed best in tissue (92%) and also remained highly accurate in blood (94%). The distinct expression of lncRNAs showed that they could play an important role in the response of UC patients to treatment. They can be a potential biomarker to monitor disease activity and predict response to anti‐TNF treatment in UC patients.

## 1. Introduction

Ulcerative colitis (UC), a type of inflammatory bowel disease (IBD), is characterized by the mucosal inflammation in the rectum to the more proximal colon, rectal bleeding, and periods of remission, and flare‐ups [1–3]. Although a series of important agents, such as exposure to toxins, changes in microbial composition, dysregulated immune responses, genetic susceptibility, and epigenetic alterations are involved in this debilitating disease, the UC pathogenesis remains to be fully identified [3–5]. Previous studies demonstrated that tumor necrosis factor (TNF)‐α is one of the most important pro‐inflammatory cytokines, which causes epithelial barrier dysfunction by altering the structure and function of tight junction (TJ) proteins in UC patients [6, 7]. Currently, anti‐TNF monoclonal antibodies (mAbs) are used to treat moderate‐to‐severe steroid‐refractory or ‐dependent UC patients [3, 8]. TNF‐α inhibitors, such as adalimumab, infliximab, and golimumab, have been widely prescribed for remitting pain and reducing inflammation in UC patients [1, 3, 6]. However, some patients with UC may experience no response to TNF‐α inhibitors, leading to a discontinuation of treatment [9]. Therefore, in addition to using the disease activity index, endoscopic observations, and histological examination, it would be helpful to identify novel, reliable, and reproducible biomarkers to predict therapeutic efficacy, prevent time consumption and disease progression, minimize side effects, and optimize personalized medicine for UC patients who are candidates to receive anti‐TNF‐α therapy [10, 11]. Several efforts have been performed to analyze the transcriptome profiles of UC patients and unravel the molecular mechanisms involved in the distinctive response to anti‐TNF therapy in pretreatment lesions of responders and nonresponders [12–15]. However, few reports have been focused to indicate the role of long noncoding RNAs (lncRNAs) as a potential discriminator in accurately determining the response of UC patients to anti‐TNF therapy in clinical practice.

LncRNAs are transcripts with more than 200 nucleotides [16] that modulate gene expression in multiple pathways, including epigenetic modification, transcription, post‐transcription, translation, and post‐translation [17–21]. Some evidence has shown changes in the expression pattern of several lncRNAs that were associated with the progression of UC via the modulation of intestinal barrier function and regulating the expression of inflammatory cytokines such as TNF‐α [21–23]. The involvement of lncRNAs in IBD pathogenesis and their potential in accurate diagnosis, prognosis, and prediction of therapeutic responses in IBD patients have been reported in previous reports [24–26].

Lucafò et al. [26] showed increased expression of the lncRNA growth arrest specific 5 (GAS5) in patients with poor response to Glucocorticoids (GCs) as well as in vitro GC‐resistant cells. Their findings indicated that GAS5 could serve as a novel pharmacogenomic marker to drive personalized GC therapy for IBD patients. Furthermore, it has been demonstrated a decrease in the expression of RNA antisense ncRNA in the INK4 locus (ANRIL) in the inflamed intestinal mucosa of Crohn’s disease (CD) patients compared to patients in the remission phase and healthy controls. This downregulation showed a significant correlation with disease activity, overexpression of inflammatory cytokines, and unresponsiveness to infliximab treatment in patients with CD [27]. Therefore, further investigation of expression changes in lncRNAs and their functional characteristics in the pathophysiology of IBD looks to be an emerging subject and a promising strategy to predict the response of UC patients to anti‐TNF therapies.

In this study, anti‐TNF‐naïve UC patients diagnosed with moderate to severe disease activity were candidates to receive treatment. We aimed to determine the potential of five lncRNAs, including GAS5, CDKN2B antisense RNA 1 (CDKN2B‐AS1), colorectal neoplasia differentially expressed (CRNDE), taurine upregulated gene 1 (TUG1), and H19 in predicting response to anti‐TNF‐α therapy in UC patients. Furthermore, their ability to monitor disease activity and inflammation was investigated by assessing their expression changes at baseline (W0) and week 14 (W14) in UC patients.

## 2. Materials and Methods

### 2.1. Study Design and Participants

Forty‐two UC patients who were naïve to anti‐TNF agents enrolled in this prospective cohort study as a discovery cohort. The recruitment criteria were as follows: (i) diagnosis of moderate‐to‐severe UC; (ii) endoscopic Mayo subscore of 2 or 3; (iii) age older than 18 years; (iv) refractory/dependent on corticosteroids; and (v) willingness to receive Adalimumab biosimilar, CinnoRa (CinnaGen, Tehran, Iran). Finally, responses to anti‐TNF treatment were evaluated in patients, according to endoscopic Mayo subscore changes, histological findings, and signs of clinical improvement at W14. Patients who had a Mayo subscore of 0 or 1 and achieved complete mucosal healing were defined as responders to anti‐TNF therapy. The whole blood and biopsy samples were collected from UC patients at baseline and W14. In addition, 22 patients with UC were also recruited as a validation cohort. This study was approved under the title “IR.SBMU.RIGLD.REC.1402.015” by the ethical review board of Research Institute for Gastroenterology and Liver Diseases (RIGLD) affiliated with Shahid Beheshti University of Medical Sciences (SBMU), (Tehran, Iran). All subjects signed informed consent documents.

### 2.2. | Hematoxylin and Eosin (H&E) Staining

Fresh colon tissue samples were fixed in 10% buffered formalin for 24 h at 4°C. After fixation, tissue specimens were embedded in paraffin, 4–5 μm‐thick sections were cut and placed on glass slides. The paraffin sections were then stained with eosin and hematoxylin for 10–20 min at room temperature (RT). Finally, the stained slides were visualized by a light microscope (20× magnification).

### 2.3. RNA Extraction and cDNA Synthesis

To assess the expression of lncRNAs in biopsies and whole blood specimens, TRIzol reagent (Invitrogen, USA) was used in order to isolate total RNA. Biopsies were ground in liquid nitrogen to achieve complete cell lysis before adding Trizol reagent. DNA contamination was removed using RNase‐free DNase (Qiagen), followed by diluting RNA with 8–10 µL RNase‐free water. Furthermore, the concentrations of total RNA were measured by NanoDrop2000 spectrophotometer (Thermo Scientific, Waltham, MA, USA). Finally, the Easy cDNA Synthesis Kit (Parstous) was used for cDNA synthesis according to the manufacturer’s protocol.

### 2.4. Quantitative Real‐Time Polymerase Chain Reaction (qRT‐PCR)

The expression levels of candidate lncRNAs were quantified by qRT‐PCR. PCR reactions were completed using SYBR Green master mix (Amplicon) in two technical replicates using a Rotor‐Gene Q System (QIAGEN). Reaction conditions of PCR were as follows: 10 s at 95°C for denaturation, followed by 40 cycles based on the calculated melting temperature (*T*_m_) of the primers for annealing, and 20 s at 72°C for primer extension. The relative expression levels of GAS5, CDKN2B, CRNDE, TUG1, and H19 were calculated by the 2^−ΔΔCt^ method, and expression levels were normalized to glyceraldehyde‐3‐phosphate dehydrogenase (GAPDH) as an internal control gene. The sequences of primers used in PCR reactions are listed in Table S1.

### 2.5. Enzyme‐Linked Immunosorbent Assay (ELISA)

The human C‐reactive protein (CRP) ELISA Kit (DCRP00, R&D Systems) was used to measure the human CRP concentration in serum of patients with UC. All operations were carried out accurately according to the manufacturer’s instructions. The CRP levels in serum were normalized to mg/L.

### 2.6. Machine Learning

In order to demonstrate the predictive potentiality of the lncRNAs to discriminate UC nonresponder (UCN) and UC responder (UCR) patients using their liquid and mucosal biopsies, the logistic regressions were applied to qRT‐PCR expression data through the three‐fold cross‐validation. Expression data were normalized to GAPDH and transformed to fold‐change values using the 2^−ΔΔCt^ method prior to analysis. Each lncRNA (H19 or TUG1) was analyzed as a single feature, so no multivariable feature selection was performed, thereby minimizing the risk of overfitting. Logistic regression was chosen as a transparent, interpretable, and low‐complexity classifier suitable for small datasets, thereby reducing the risk of overfitting. For model evaluation, stratified three‐fold cross‐validation was used, in which the dataset was partitioned into three equally sized folds while preserving the original responder/nonresponder ratio in each fold. In each cross‐validation iteration, two folds were used for training and the remaining fold served as an independent test set. All preprocessing steps were applied strictly within the training fold to avoid any data leakage into the test fold.

Performance metrics (accuracy, sensitivity, and specificity) were computed in each test fold and then averaged across folds to obtain an unbiased estimate of model performance. For threshold optimization, Youden’s J statistic was used to find an optimal decision threshold that maximizes sensitivity and specificity and subsequently applied unchanged to the validation cohort. For the test cohort, we trained a logistic regression model on all discovery samples and applied it to the entire independent test dataset.

The receiver operating characteristic (ROC) curve was constructed for each condition, and the area under the ROC curve (AUC) was calculated to visualize the predictive performance of the models using the R package pROC. ROC curves plot sensitivity vs. 1 − specificity over all thresholds. AUC is threshold independent and measures overall discrimination. The AUC values include 95% confidence intervals derived from DeLong.

### 2.7. Correlation

Spearman correlation test was employed to evaluate the correlation of H19 and TUG1 expression levels with the levels of erythrocyte sedimentation rate (ESR), CRP, age, sex, disease duration, endoscopic Mayo scores, white blood cell (wbc) count, platelet (PLT) count, smoking, serum iron, ferritin, vitamin D3, and hemoglobin (Hb).

### 2.8. Statistical Analysis

This study is hypothesis‐driven rather than exploratory in which five candidate lncRNAs were selected based on previous biological evidence and published literature. Statistical comparisons were, therefore, limited to a small, predefined set of targets rather than a large‐scale screening, which substantially reduces the risk of inflated type I error. Data analyses were performed using GraphPad Prism 9 software. Five lncRNAs expression data were prepared as (mean ± standard error mean [SEM]) and compared between two groups, UCRs and UCNs, using an unpaired *t*‐test. Furthermore, the paired *t*‐test was applied to compare W14 with the baseline data in the UCR or UCN group. *p* < 0.05 was considered statistically significant. Group sizes in the discovery cohort fall within a range where *t*‐tests are considered robust, particularly given the moderate‐to‐large effect sizes observed. Moreover, *t*‐tests are widely used and accepted for qRT‐PCR–based expression comparisons in studies with similar sample sizes. The key findings derived from these tests were further tested by complementary, assumption‐independent analyses, including ROC curve analysis with DeLong confidence intervals and cross‐validated logistic regression models, which do not rely on normality assumptions.

## 3. Results

### 3.1. Clinical and Histopathological Characteristics of Patients

A cohort, including 42 anti‐TNF‐naive UC patients with moderate‐to‐severe active disease started anti‐TNF‐α treatment and were followed over 14 weeks as a discovery group. Based on endoscopic findings, histopathological results and clinical outcomes after 14 weeks, UC patients were divided into UCR (*n* = 22) and UCN (*n* = 20) to the anti‐TNF‐α mAb. Comparison of colonoscopy findings between UC patients at baseline and W14 showed more severe and extensive inflammation among nonresponders at W14 (endoscopic Mayo 2 or 3) (Figure 1A1–B2, D1, D2). In contrast, there was no evidence of blood, erosion, or ulceration in the colonic mucosa of UCR patients after anti‐TNF therapy at W14 (Figure 1C1, C2). Histopathological examination revealed neutrophil infiltration, crypt abscesses, erosion, and ulceration in UCR and UCN specimens at baseline (Figure 1E1–F2), indicating disease severity and intestinal damage in these patients before receiving anti‐TNF. Furthermore, microscopic features showed histologic healing with the absence of inflammation in UCR patients after anti‐TNF treatment at W14 (Figure 1G1, G2), while no evidence of improvement was observed in nonresponders at W14 (Figure 1H1, H2). The demographic characteristics and clinical information of patients with UC at baseline are reported in Table 1. The mean age of UCRs and UCNs was 33.55 ± 12.02 and 30.83 ± 11.26 years, respectively. The numbers of female and male patients were 11 (50%) and 11 (50%) in UCR and 7 (35%) and 13 (65%) in UCN, respectively. The median disease duration of responders was 66.94 ± 54.67 months and 44.22 ± 41.24 months for UCN. Furthermore, 22 UC patients (12 UCR and 10 UCN) were also included in the validation group. The clinical characteristics of patients with UC at W0 were presented in Table S2.

Figure 1Colonoscopic and histopathological findings in UCR and UCN patients. Colonoscopy results of UCR (A1, A2) and UCN patients (B1, B2) at W0. Colonoscopy results of UCR (C1, C2) and UCN (D1, D2) patients at W14. Histopathological results of UCR (E1, E2) and UCN (F1, F2) patients at W0. Histopathological results of UCR (G1, G2) and UCN (H1, H2) patients at W14 refers to the ulcerative colitis responders and UCN refers to the ulcerative colitis nonresponders to anti‐TNF therapy.

**Figure:**
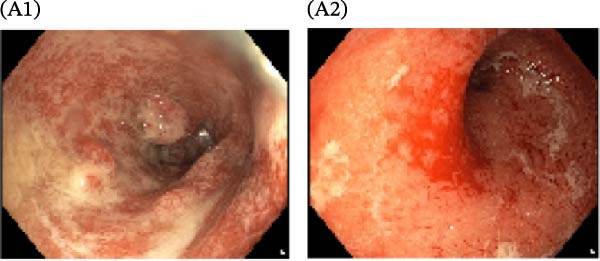

**Figure:**
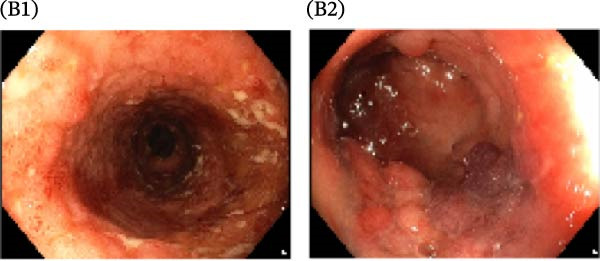

**Figure:**
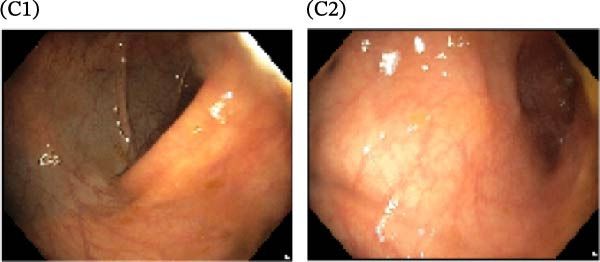

**Figure:**
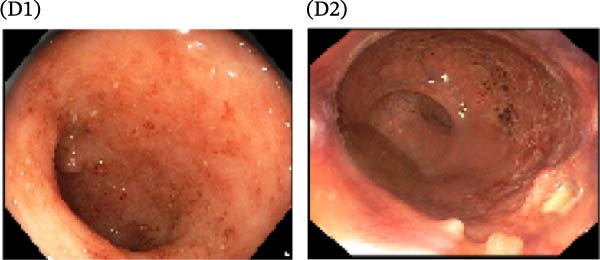

**Figure:**
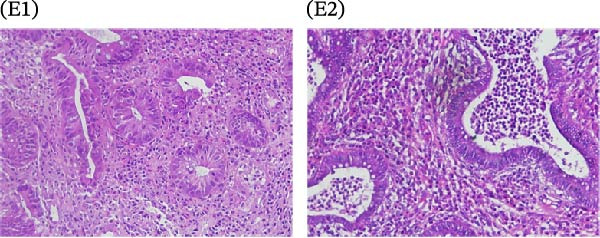

**Figure:**
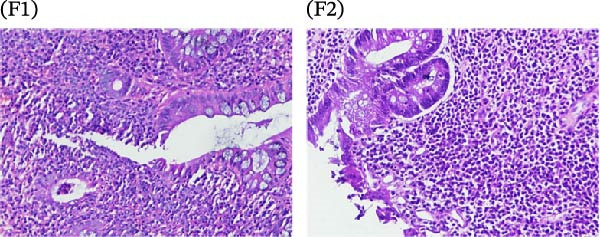

**Figure:**
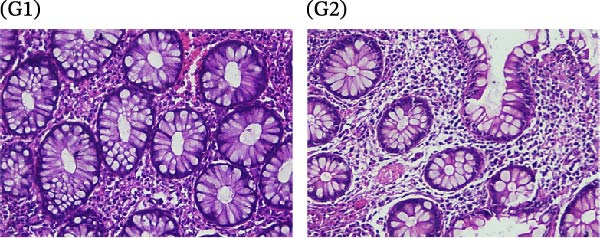

**Figure:**
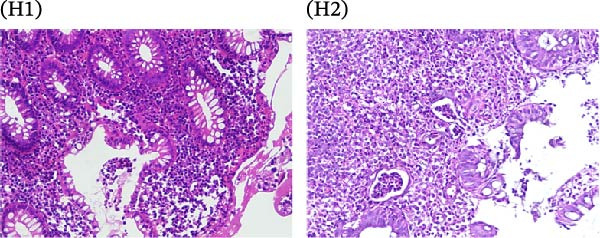

**Table 1:** Basic clinical characteristics of UC responders and nonresponders to anti‐TNF‐α treatment in the discovery cohort.

Variables	UCR (*N* = 22)	UCN (*N* = 20)
Gender		
Female	11 (50%)	7 (35%)
Male	11 (50%)	13 (65%)
Age (year)	33.55 ± 12.02	30.83 ± 11.26
Family history	—	1 (10%)
Smoking		
Smoker	13 (59%)	6 (30%)
Non‐smoker	9 (41%)	14 (70%)
Disease duration (month)	66.94 ± 54.67	44.22 ± 41.24
ESR (mm/h)	33.69 ± 21.4	34.57 ± 17.16
CRP (mg/L)	17.18 ± 16.4	30.83 ± 30.14
Hb (g/dL)	11.62 ± 2.68	11.91 ± 2.29
WBC (10^3^/μL)	7.53 ± 3.07	8.03 ± 0.93
MCV (femtoliters [fL])	87.2 ± 5.94	88.5 ± 6.1
PLT (10^4^/μL)	39.17 ± 12.94	33.92 ± 9.22
Serum Iron (μg/dL)	39.6 ± 38.42	23.66 ± 15.17
Ferritin (ng/mL)	55.67 ± 37.53	60.5 ± 65.5
Vit D3 (ng/mL)	29.31 ± 14.60	22.4 ± 8.51

### 3.2. Distinct Expression Pattern of lncRNAs in UCN Lesions Compared to UCR Ones

We evaluated the expression of five lncRNAs in the inflamed biopsies of UCN and UCR patients prior to receiving anti‐TNF therapy. The relative expression levels of these lncRNAs showed that H19 and TUG1 were significantly dysregulated in UCN compared to UCR. H19 expression was significantly upregulated in the inflamed lesions of UCN patients compared to UCR (Figure 2A), while the expression level of TUG1 was significantly lower than in patients with responsiveness (Figure 2B). However, we found no differences in the expression of GAS5, CDKN2B‐AS1, and CRNDE between UCN and UCR at baseline (Figure 2C–E). The Spearman test was used to evaluate the correlation between the expression of lncRNAs (H19 and TUG1) and the basic clinical characteristics of UC patients, including age, sex, disease duration, endoscopic Mayo scores, CRP, ESR, WBC, PLT, smoking, serum iron, ferritin, vitamin D3, and Hb. Our findings indicated a significant positive correlation between the expression of H19 and the levels of CRP and ESR as two hallmarks of inflammation. Furthermore, expression levels of H19 and TUG1 were positively correlated with endoscopic Mayo scores with *r* approximately 0.70 and *p* approximately 0.0037 for H19, and *r* approximately 0.55 and *p* approximately 0.035 for TUG1, suggesting that circulating lncRNA signal may reflect subsequent disease activity. However, we did not observe significant correlations between lncRNA expression levels and disease duration, age, sex, WBC, PLT, smoking, serum iron, ferritin, vitamin D3, and Hb in our dataset. The full correlation table is provided in Table S3.

Figure 2Expression of five lncRNAs in tissue biopsies of UCN and UCR patients at baseline. Normalized expression levels of (A) H19, (B) TUG1, (C) GAS5, (D) CDKN2B‐AS1, and (E) CRNDE in the inflamed lesions of UC patients before anti‐TNF therapy at W0.  ^∗^ *p* < 0.05,  ^∗∗^ *p* < 0.01. UCR refers to the ulcerative colitis responders and UCN refers to the ulcerative colitis nonresponders to anti‐TNF therapy.

**Figure:**
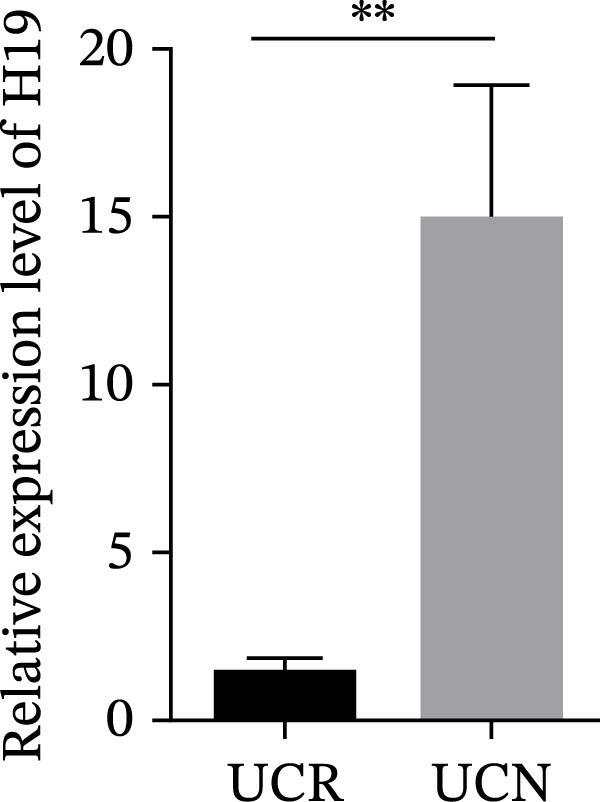
(A)

**Figure:**
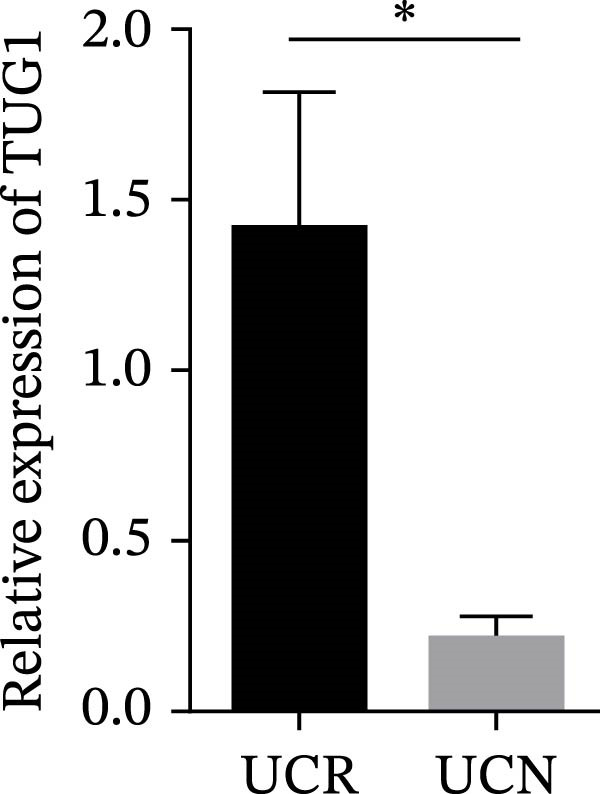
(B)

**Figure:**
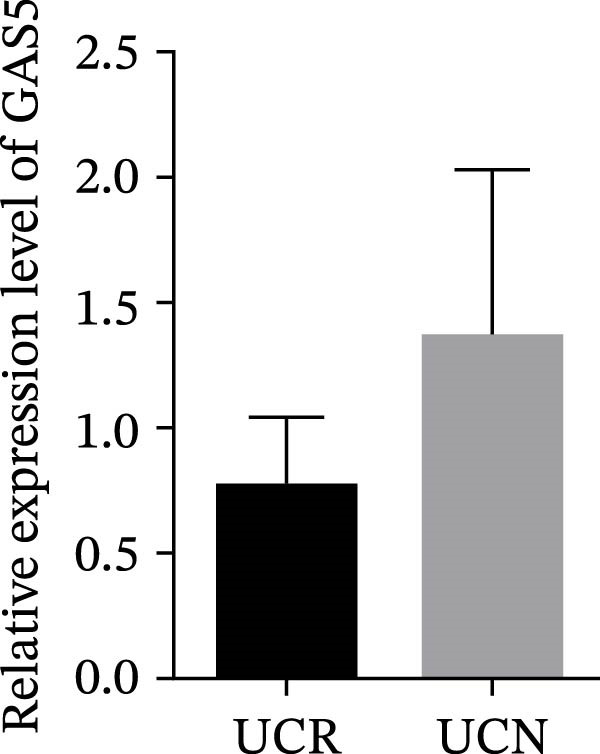
(C)

**Figure:**
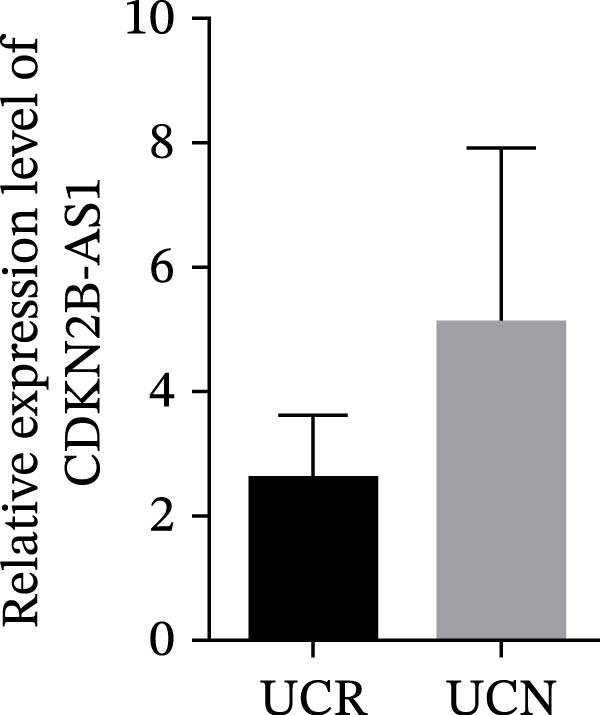
(D)

**Figure:**
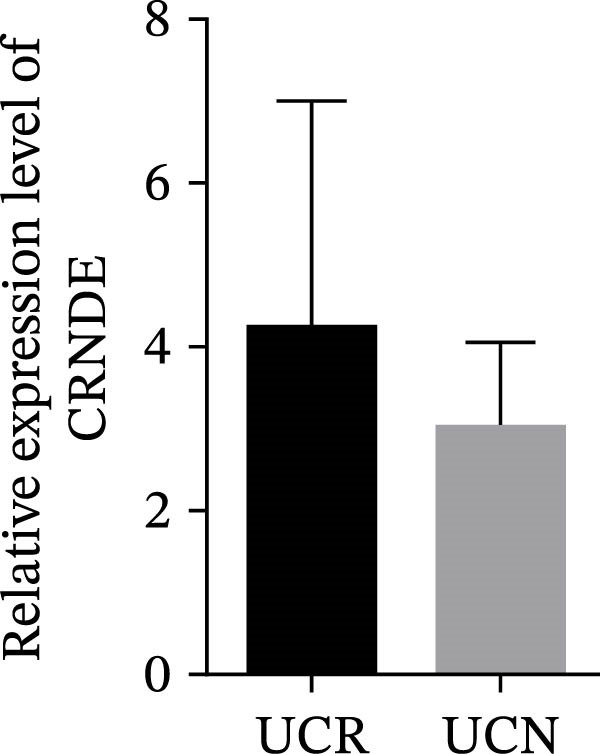
(E)

The expression of two lncRNAs was re‐evaluated at W14 after the beginning of treatment in both groups of UC patients. Intriguingly, results showed that H19 expression was still significantly higher in UCN at W14 compared to UCR (Figure 3A), while no significant difference was observed in the expression level of TUG1 between UCR and UCN at W14 (Figure 3B). Therefore, upregulation of H19 is likely to be involved in the distinctive molecular response of UC patients to anti‐TNF mAbs.

Figure 3Expression of H19 and TUG1 in tissue biopsies of UCN and UCR patients at W14. (A) H19 and (B) TUG1.  ^∗∗^ *p* < 0.01.

**Figure:**
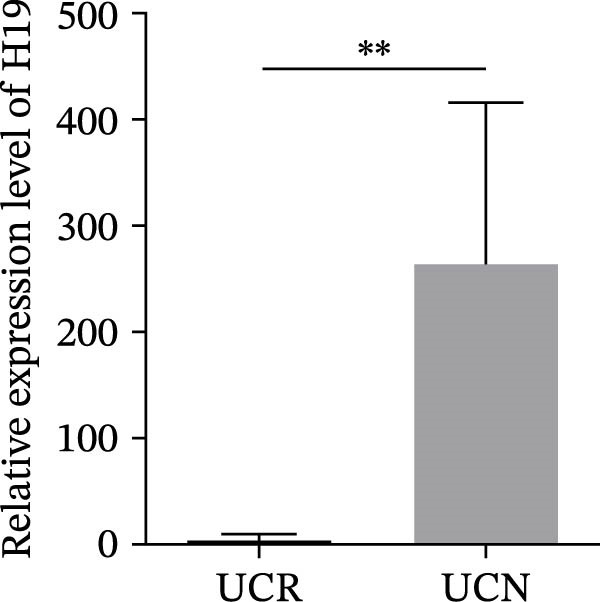
(A)

**Figure:**
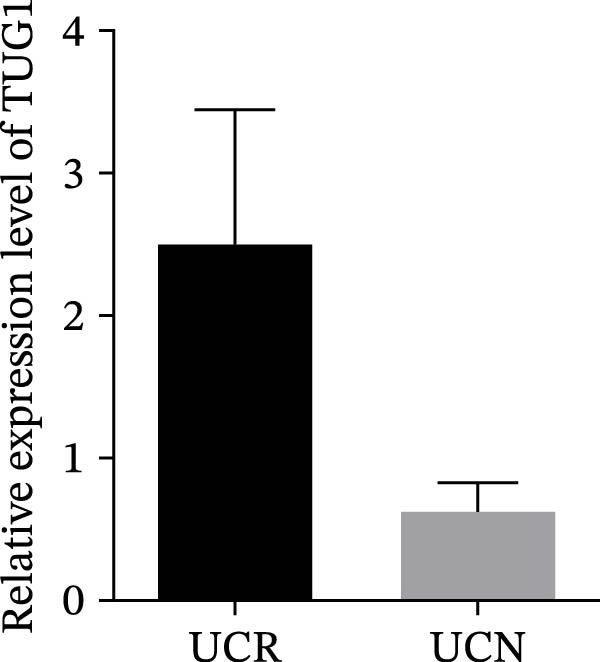
(B)

### 3.3. The Effect of Anti‐TNF Therapy on the Colonic Expression of lncRNAs in UC Patients

To further evaluate the effect of anti‐TNF mAb, the expression of H19 and TUG1 at W14 was compared with baseline. Our results demonstrated that the transcript level of H19 was significantly reduced in the colon tissue of UCR after anti‐TNF treatment (Figure 4A). Intriguingly, a decrease was also observed in TUG1 expression in UCR samples at W14 (Figure 4B). Therefore, lncRNAs can play important roles in reducing inflammation, inducing remission and managing disease activity in UCR.

Figure 4Changes in the colonic expression of H19 and TUG1 after receiving anti‐TNF mAb. (A) Relative expression levels of H19 at W0 and W14 in UCR patients. (B) Relative expression levels of TUG1 at W0 and W14 in UCR patients. (C) Relative expression levels of H19 at W0 and W14 in UCN. (D) Relative expression levels of TUG1 at W0 and W14 in UCN patients.  ^∗∗^ *p* < 0.01.

**Figure:**
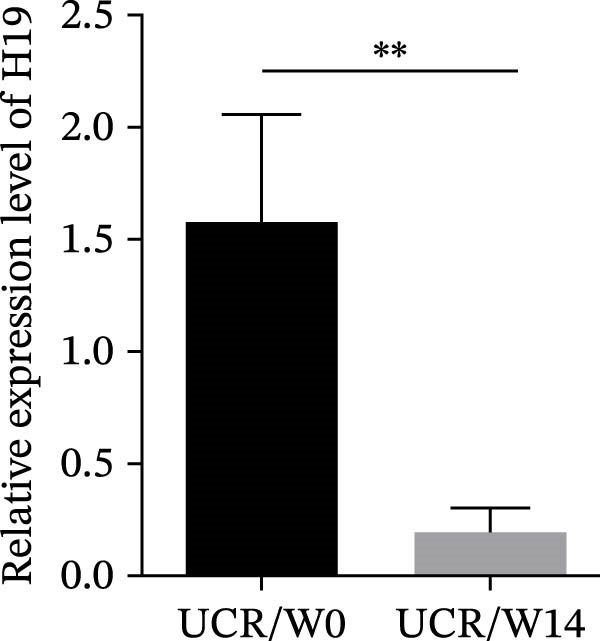
(A)

**Figure:**
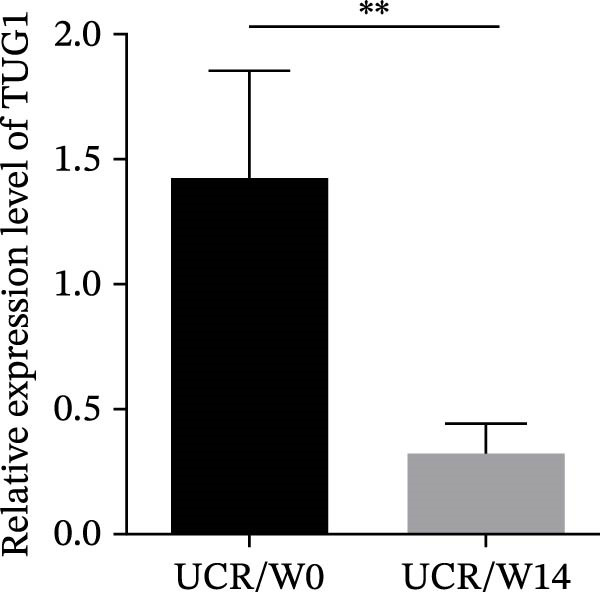
(B)

**Figure:**
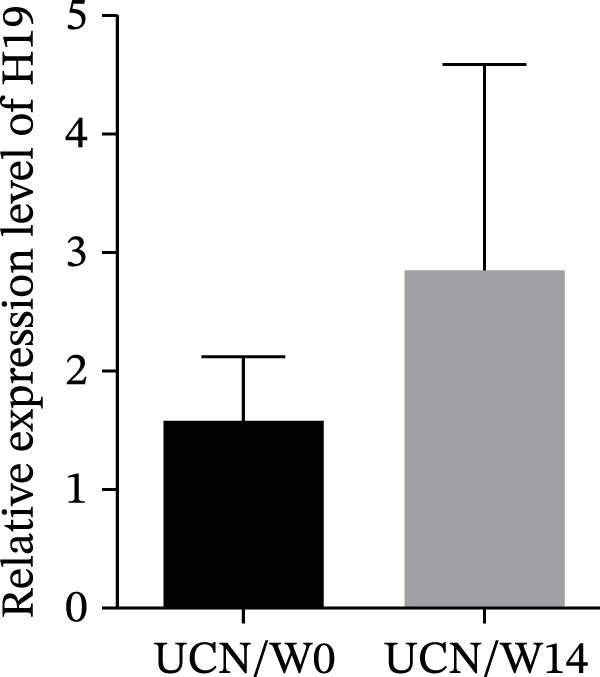
(C)

**Figure:**
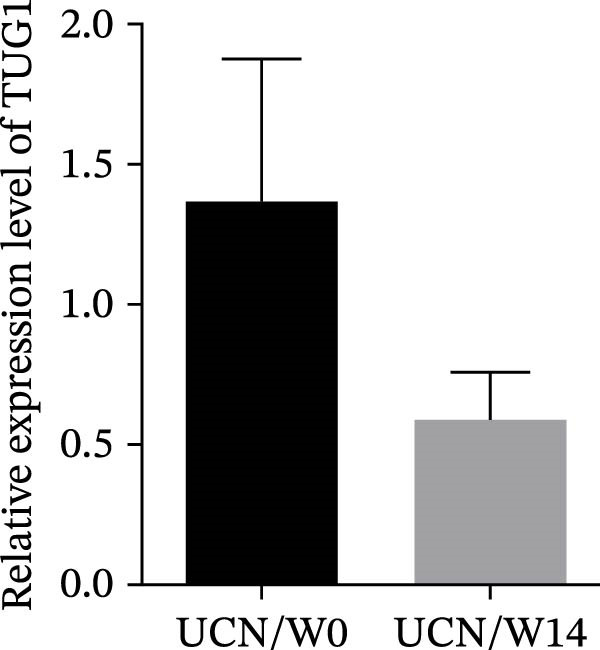
(D)

Likewise, we analyzed the expression of lncRNAs in UCN samples. The expression of both H19 and TUG1 showed no significant changes in UCN after anti‐TNF therapy (Figure 4C, D).

### 3.4. Distinct Expression Pattern of lncRNAs in Whole Blood Samples of UCN Patients Compared to UCR Ones

The expression levels of H19 and TUG1 were also evaluated in the whole blood samples of UC patients, prior to receiving anti‐TNF therapy. Results showed that the expression level of H19 increased in UCN patients compared to UCR at baseline, similar to what was observed in biopsies (Figure 5A), while unlike the expression in tissues, TUG1 was significantly upregulated in whole blood samples of UCN patients compared to UCR patients (Figure 5B). Furthermore, the expression levels of these two lncRNAs were re‐evaluated in these patients at W14 after the start of treatment. Similarly, the expression levels of both H19 and TUG1 were significantly higher in UCN compared to UCR patients at W14 (Figure 5C, D).

Figure 5Whole blood expression of H19 and TUG1 in UCN and UCR patients at W0 and W14. Comparison of H19 (A) and TUG1 (B) expression in whole blood samples of UCN and UCR patients at W0. Comparison of H19 (C) and TUG1 (D) expression in the whole blood samples of UCN and UCR patients at W14.  ^∗^ *p* < 0.05.

**Figure:**
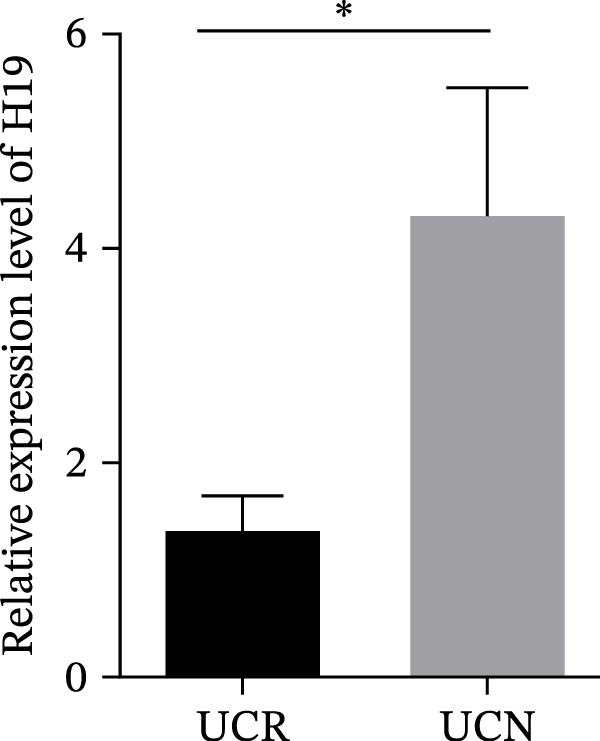
(A)

**Figure:**
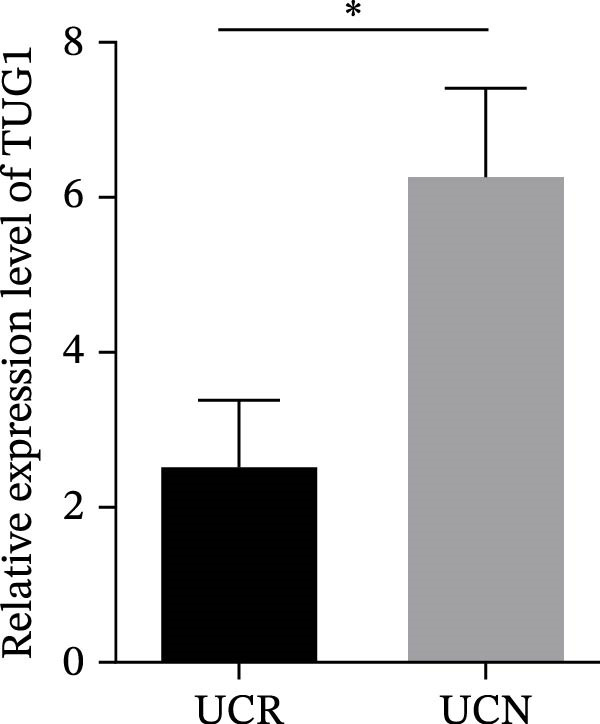
(B)

**Figure:**
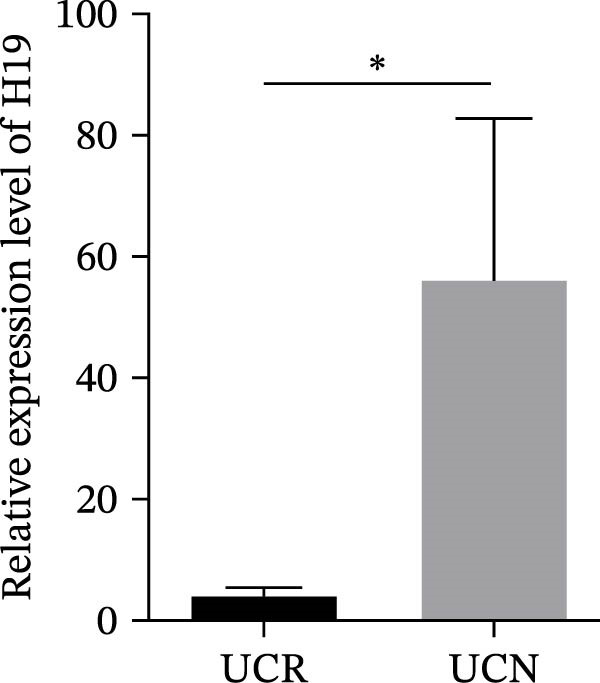
(C)

**Figure:**
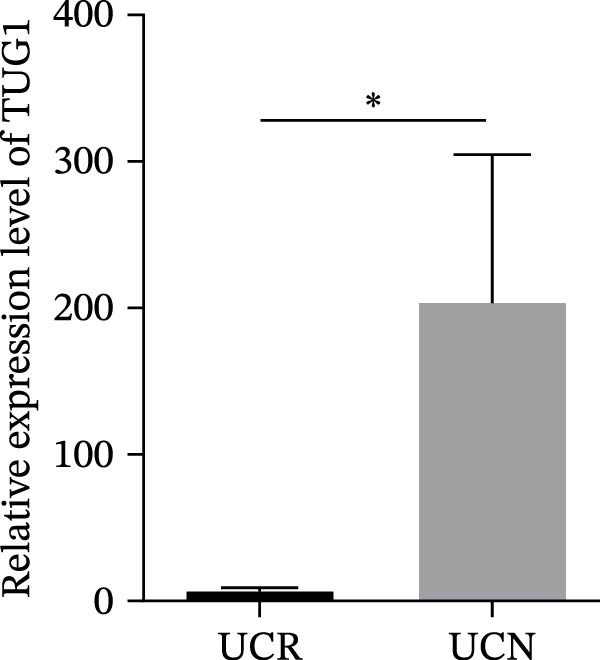
(D)

### 3.5. Considerable Performance of lncRNAs in Predicting Response of UC Patients to Anti‐TNF Therapy

To evaluate the predictive power of lncRNAs for the response of UC patients to anti‐TNF therapy, logistic regression was performed based on the three‐fold cross‐validation method. Our findings showed that both H19 and TUG1 exhibit strong predictive value for distinguishing UCNs from UCRs to anti‐TNF therapy. The H19 expression in tissue and blood revealed a high predictive accuracy of 90% (sensitivity 83% and specificity 100%) and 85% (sensitivity 92% and specificity 83%), respectively. This suggests that H19 expression is consistently altered in nonresponders across compartments, making it a reliable marker. TUG1 performed exceptionally well in tissue (accuracy 93%; sensitivity 100%, specificity 89%), but showed much lower accuracy in blood (60%).

Furthermore, ROC curves which were sketched to identify biomarkers with higher AUC represented a strong discriminative capacity for TUG1 in tissue with AUC of 0.88 (95% CI: 0.82–1) (Figure 6A). Likewise, the ROC curve for TUG1 in blood (Figure 6B) and H19 in tissue and blood (Figure 6C,D) represented noticeable AUCs of 0.84 (95% CI: 0.71–0.95), 0.84 (95% CI: 0.78–1), and 0.83 (95% CI: 0.72–0.97), respectively. These are considered good classifier values, indicating robust performance across markers. Collectively, these results suggest that TUG1 (tissue) is the strongest single predictor, whereas H19 performs more consistently between tissue and blood.

Figure 6ROC curves for predicting response to anti‐TNF mAb treatment at baseline. The ROC curves showed the performance of (A, B) TUG1 and (C, D) H19 biomarkers in diagnosing UCN from UCR before receiving anti‐TNF therapy using tissue and whole blood samples, respectively.

**Figure:**
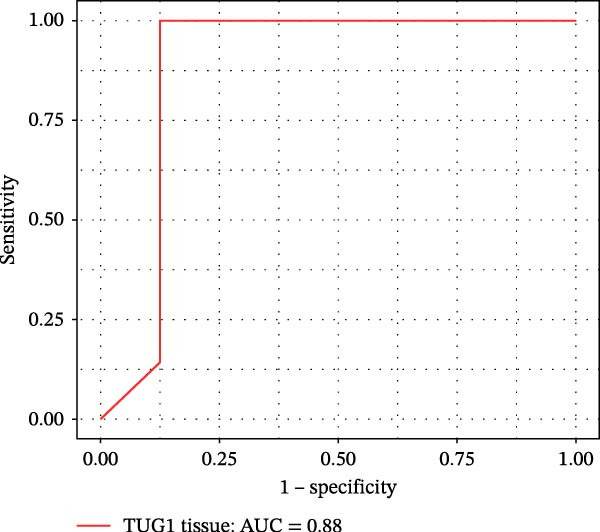
(A)

**Figure:**
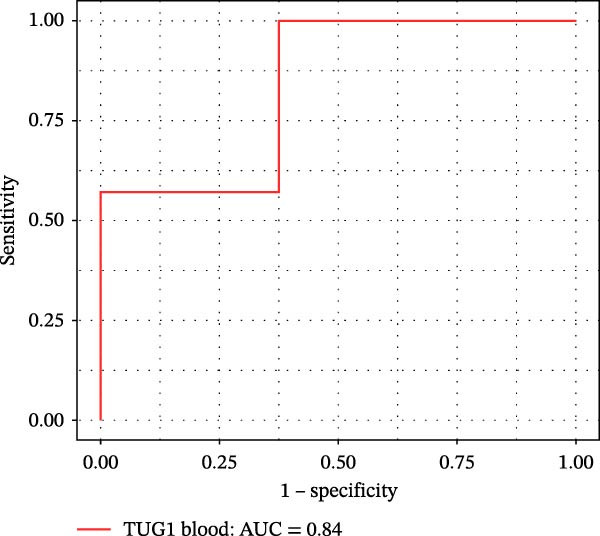
(B)

**Figure:**
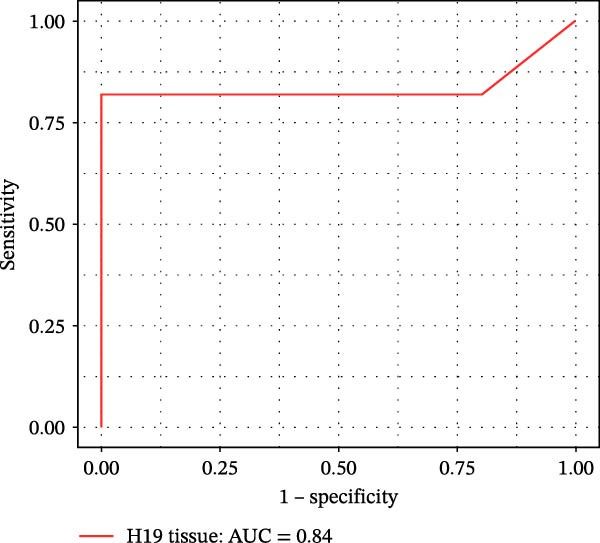
(C)

**Figure:**
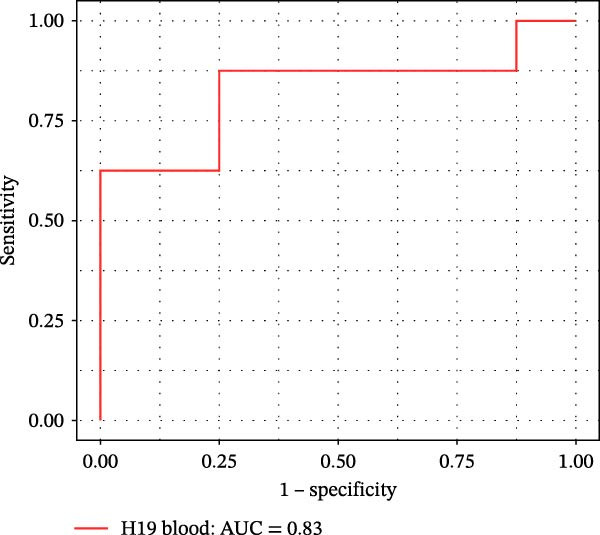
(D)

To authenticate the expression changes and discriminative potentiality of the predictive markers, qRT‐PCR was used for mucosal biopsies and blood samples of an independent patient cohort (10 UCN and 12 UCR). The independent validation cohort reproduced the same expression trends. H19 was significantly upregulated in mucosal biopsies of UCN patients (Figure 7A) while TUG1 was significantly downregulated (Figure 7B). Blood expression patterns at baseline also matched the discovery cohort, showing increased H19 and TUG1 levels in nonresponders (Figure 7C,D). This consistency supports the biological robustness of the lncRNA signatures and indicates that these biomarkers are not cohort‐dependent.

Figure 7The predicting performance of H19 and TUG1 in the validation cohort. Relative expression of (A) H19 and (B) TUG1 in colonic biopsies of UCN and UCR of the validation cohort. Relative expression of (C) H19 and (D) TUG1 in whole blood samples of UCR and UCN of the validation cohort.  ^∗^ *p* < 0.01. The ROC curves indicated the performance of (E, F) H19 and (G, H) TUG1 biomarkers in predicting response to anti‐TNF therapy in the validation cohort.

**Figure:**
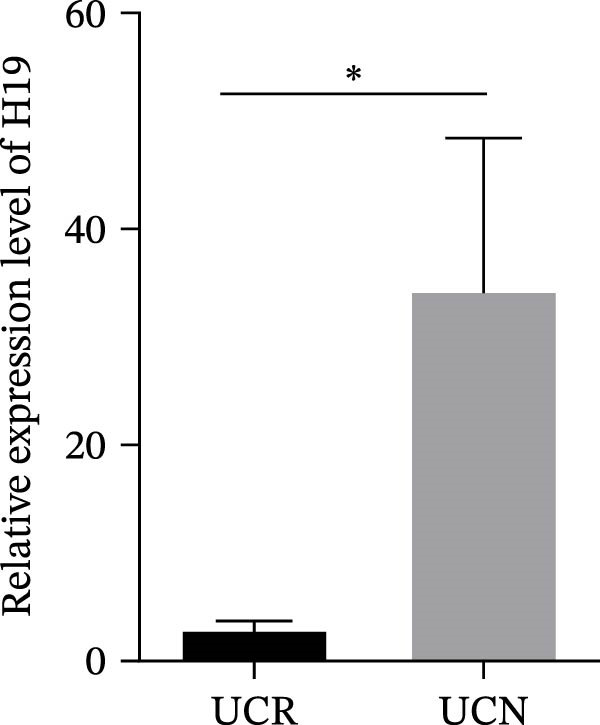
(A)

**Figure:**
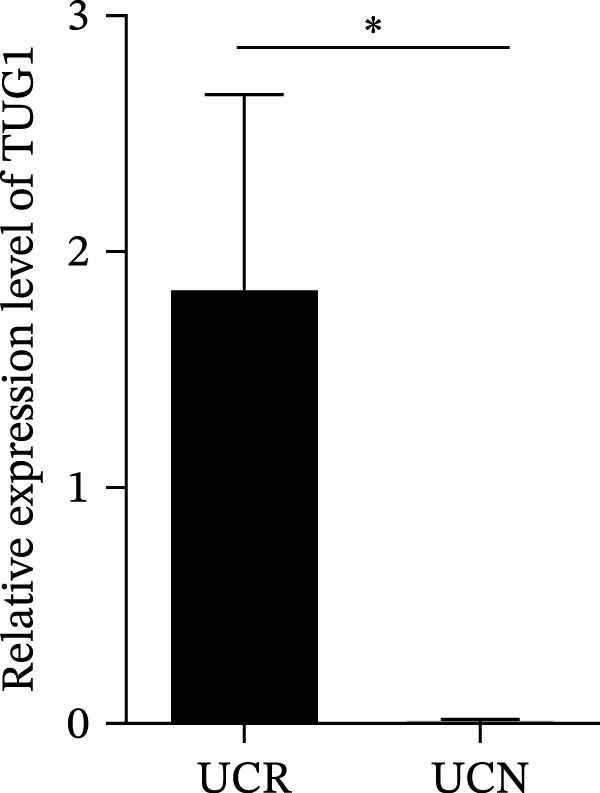
(B)

**Figure:**
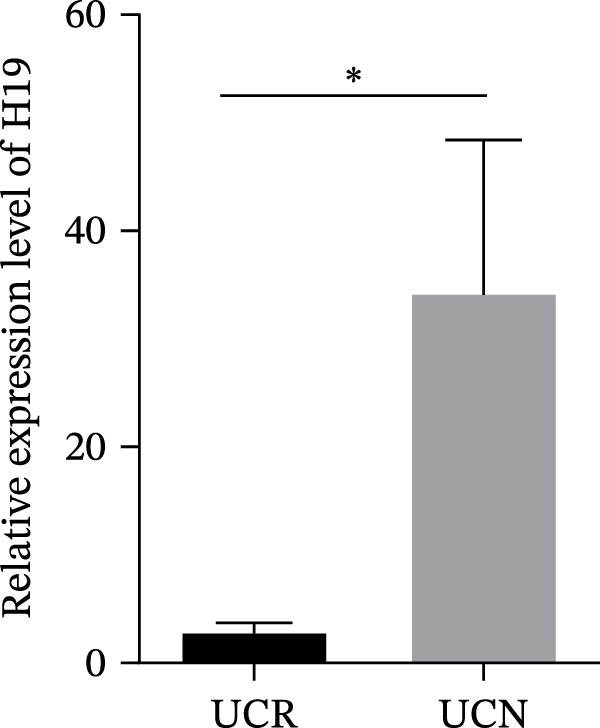
(C)

**Figure:**
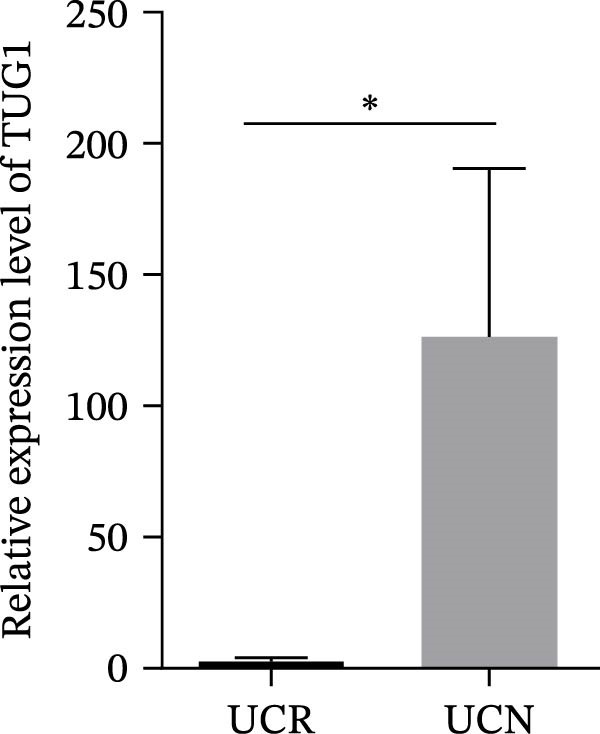
(D)

**Figure:**
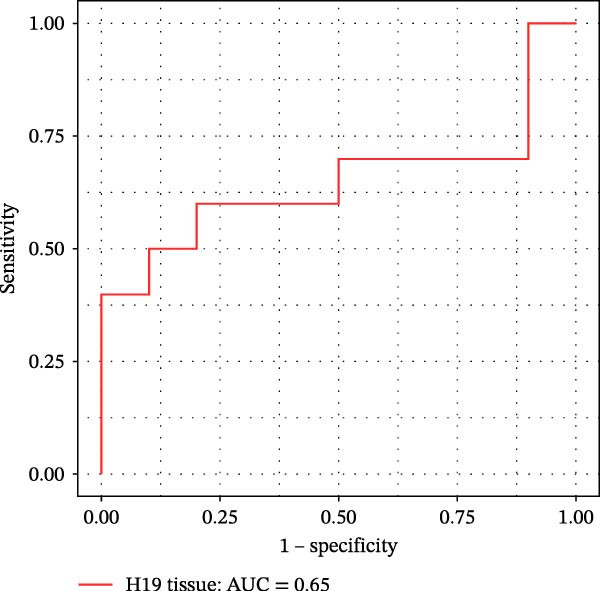
(E)

**Figure:**
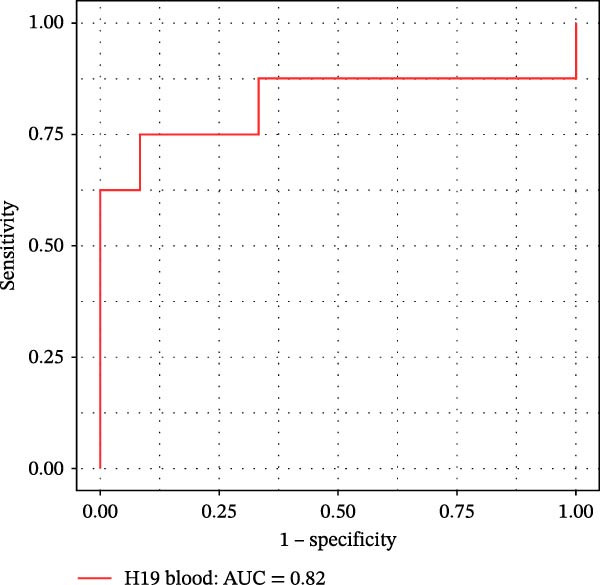
(F)

**Figure:**
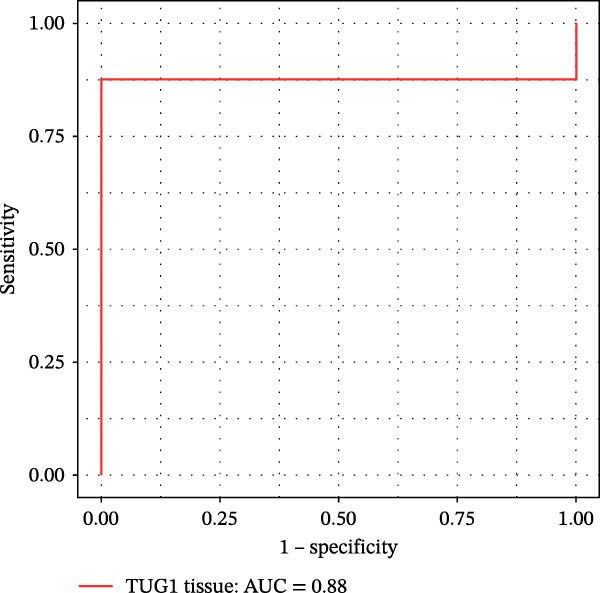
(G)

**Figure:**
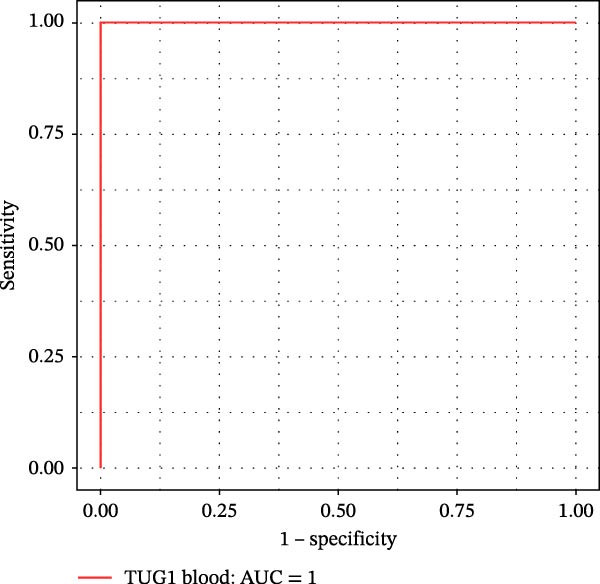
(H)

The trained logistic regression classifier also performed well on validation samples. H19 in tissue samples showed a moderate accuracy (70%) and modest AUC (0.65, 95% CI: 0.49–0.98), indicating a reduced predictive strength in validation that is common in real‐world cohorts where tissue heterogeneity is high, while it had a strong performance (85% accuracy, AUC 0.82, 95% CI: 0.6–1) in blood, suggesting that blood‐based H19 could be a practical and reliable clinical marker (Figure 7E,F). TUG1 demonstrated an excellent performance in tissue (92% accuracy, AUC 0.88, 95% CI: 0.73–1) and a near‐perfect performance (94% accuracy, AUC 1.00, CI of a ROC curve with AUC = 1 is always 1–1 and can be misleading) in blood samples (Figure 7G,H). These results reinforce the observation that TUG1 in tissue is consistently the most accurate predictor across both discovery and validation datasets, whereas H19 could be more useful as a blood‐based biomarker, offering a less invasive option for clinical use.

## 4. Discussion

IBD therapy and diagnosis confront many challenges, which can only be addressed by acquiring a better knowledge of the molecular pathways involved in disease development. On the other hand, lncRNAs, which have been proven to be novel and powerful players in gene regulation, have also recently been investigated in IBD [24]. Considering the important roles of lncRNAs in the pathogenesis of UC, we explored the relationship between lncRNAs, and the response to anti‐TNF‐α treatment. We studied the expression patterns of lncRNAs among two groups of anti‐TNF‐naive UC patients (UCR and UCN) prior to treatment to understand whether lncRNAs can play a role in the different responses of UC patients to anti‐TNF therapy. Our results demonstrated that the expression pattern of H19 and TUG1 in UCN compared to UCR were significantly different at baseline, while no alterations were found in the GAS5, CDKN2B‐AS1, and CRNDE expression. Furthermore, we focused on the alteration of H19 and TUG1expression during anti‐TNF therapy in UC patients to investigate the impact of treatment on the expression of lncRNAs, leading to better understanding of H19 and TUG1 functions in disease activity and managing inadequate response to anti‐TNF therapy in UC patients.

H19 has been identified as an important lncRNA in the pathogenesis of patients with UC [28]. It has been reported to be involved in the immune response process and barrier dysfunction of epithelial cells induced by pathogens [29]. Likewise, H19 expression has been associated with intestinal barrier impairment and induced epithelial mesenchymal transition (EMT) in UC patients [30, 31]. Another study showed that H19 overexpression may induce a destructive effect on intestinal epithelial barrier function through downregulation of vitamin D receptor (VDR) signaling in UC patients, suggesting that H19 can play a critical role in the development of UC [32]. It has been reported that the pathophysiology of UC is linked to the high expression of the inflammatory cytokine TNF‐α, leading to inflammatory damage. Indeed, TNF‐α stimulates the expression of inflammatory cytokines through increased expression of H19 [33]. Studies have also shown that H19 deletion reduced apoptosis and the production of pro‐inflammatory molecules, such as TNF‐α, interleukin (IL)‐1, and IL‐6, while H19 overexpression activated the nuclear factor kappa B (NF‐κB) signaling pathway and enhanced the production of inflammatory factors [34, 35]. Furthermore, silencing H19 has been observed to be associated with the expression of IL‐1beta (IL‐1β) and TNF‐α in UC mice [30]. LncRNA H19 has been shown to be overexpressed in dextran sodium sulfate (DSS)‐induced UC mouse model. Silencing H19 reduced disease severity, decreased proinflammatory cytokines, such as TNF‐α and IL‐1β, increased the level of anti‐inflammatory cytokine, IL‐10, and also improved histopathological features. Mechanistically, lncRNA H19 acts as an aggravating factor of intestinal injury through the miR‐331‐3p/TNF receptor‐associated factor 4 (TRAF4) axis [30]. Furthermore, the strong association between H19 and TNF‐α expression levels was observed in several disorders, such as rheumatoid arthritis (RA) [36], bronchopulmonary dysplasia (BPD) [37], and acute kidney injury (AKI) [38]. It has been shown that the expression level of lncRNA H19 was increased by TNF‐α stimulation in RA synovial cells (MH7A cells), leading to increased inflammatory cytokines through the TAK1/NF‐κB JNK/p38 MAPK signaling pathways. Whereas, silencing of H19 reversed these effects [36]. These findings indicate that H19 can modulate inflammatory signaling pathways commonly activated by TNF‐α.

In our previous study, we showed that H19 expression was decreased in IBD patients in the remission phase compared to active IBD patients, highlighting its positive relationship with disease activity [25]. Our search through the Cui Lab/LncRNADisease database (http://www.cuilab.cn/lncrnadisease), and miRNet 2.0 (www.mirnet.ca/miRNet/Secure/NetworkBuilder.xhtml) also showed considerable interactions between two lncRNAs (H9 and TUG1) and important inflammatory genes, particularly TNF‐α. However, little was known about the distinguishing expression pattern of H19 in UCR and UCN patients as well as its contribution to inflammation suppression and decreased disease activity after anti‐TNF therapy. Our results demonstrated that H19 expression is upregulated in both whole blood and biopsy samples of UCN compared to UCR patients at the baseline and W14, and its high expression is positively correlated with elevated inflammation markers (including CRP and ESR). Therefore, H19 appears to be involved in a more severe inflammatory condition in UCN and can serve as a predictive biomarker to determine nonresponsiveness to anti‐TNF treatment in UC patients at baseline. In a more profound analysis, we compared the expression level of H19 in colon biopsies of UCR at baseline and W14 after receiving anti‐TNF and found that its expression was decreased after treatment with anti‐TNF. However, H19 expression in UCN at W14 after treatment did not show a significant difference compared to the baseline. Therefore, our results showed that anti‐TNF therapy may alleviate disease symptoms in UCR by reducing H19 expression. These findings strongly imply that H19 is an inflammatory lncRNA associated with response to anti‐TNF drugs in biopsy and whole blood samples of UCR.

TUG1 is a long intergenic noncoding RNA that regulates gene expression through chromatin reprogramming [39]. The development of some human illnesses is thought to be significantly associated with TUG1 dysregulation [40–42]. It has been reported that TUG1 was downregulated in colonic mucosa tissues of UC patients [43]. It has been identified that TUG1 overexpression inhibited UC progression and apoptosis in TNF‐α‐treated IECs by regulating the balance of HuR and miR‐29b 3p [44]. Also, TUG1 can inhibit TNF‐α‐induced intestinal epithelial cell death by increasing the cyclin‐dependent kinase 2 (CDK2) expression [45]. In addition, TUG1 prevents the production of TNF‐α‐induced inflammatory cytokines, including IL‐6, IL‐8, and IL‐1β in TNF‐α‐treated HT‐29 cells by regulating the miR‐142‐5p/suppressor of cytokine signaling 1 (SOCS1) axis [22]. Previous studies have also proven that upregulation of TUG1 causes a decrease in TNF‐induced inflammation via reducing miR‐127 and inhibiting NOTCH signaling [46]. Furthermore, strong evidence also suggests a link between TUG1 and TNF‐α expression levels in multiple disorders, such as irritable bowel syndrome (IBS) [46], sepsis [47], and osteoporosis [48]. These findings thus imply that TUG1 may be involved in IEC apoptosis and UC development through several molecular mechanisms. To demonstrate the contribution of TUG1 to response to the anti‐TNF drug, here we showed that the expression of TUG1 was lower in biopsy samples of UCN than UCR at baseline and W14, while it showed an overexpressed pattern in whole blood of UCN compared to UCR patients at baseline. Based on the aforementioned results, it seems that patients with UC have a distinct expression pattern of TUG1 in blood and colon tissue depending on their response to anti‐TNF therapy.

One‐third of IBD patients do not respond to anti‐TNF therapy which may be associated with potentially severe systemic side effects [49]. Therefore, finding biomarkers to predict therapy responsiveness and clinical outcomes is important in UC patients. A recent study revealed that in patients with active CD, lnc‐ITSN1‐2 expression was reduced following TNF inhibitor treatment, indicating it may be used as a possible biomarker to predict the effectiveness of TNF inhibitor therapy [50]. In this study, a logistic regression approach was used to perform a classification analysis for the prognosis of responders and nonresponders based on the expression of lncRNAs in biopsy and blood samples. We demonstrated that the expression of H19 and TUG1 in UC patients’ colon biopsies could predict the response to anti‐TNF medication with 90% and 93% accuracy at baseline in the discovery cohort, respectively. Recruiting and phenotyping a treatment cohort for anti‐TNF response is resource‐intensive. It requires endoscopy or standardized clinical assessment, strict timing relative to drug exposure, and harmonized biospecimen collection for both tissue and blood. Building such a dataset at a single center could be challenging and time‐consuming. To reduce optimism in estimates and obtain generalizable results, we used three‐fold cross‐validation with balanced folds and we limited model complexity to single‐marker models. These choices provide internally consistent estimates of discrimination while acknowledging the limits of sample size. Furthermore, the validation experiments strengthened the biomarker candidates by confirming that expression trends were not limited to the discovery population. Of note, further validation in a larger independent cohort can provide us with more reliable findings and prevent misleading results. Differential expression of H19 and TUG1 was observed consistently in both mucosal biopsies and peripheral blood, and logistic regression models trained on the discovery cohort maintained strong classification performance when applied to the independent dataset. Both H19 and TUG1 exhibited strong discriminative performance for predicting anti‐TNF response in UC. TUG1, particularly in mucosal tissue, consistently showed the highest accuracy and AUC values, indicating its strong potential as a tissue‐based predictive biomarker. H19 demonstrated a robust performance across both tissue and blood, highlighting its potential utility as a minimally invasive blood‐based biomarker. These results not only emphasize the reproducibility of the lncRNA signatures but also highlight their potential utility in real‐world clinical settings where patient variability is high. Collectively, our findings suggest that TUG1 and H19 may provide clinically meaningful tools for early identification of UC patients unlikely to respond to anti‐TNF therapy, enabling more personalized treatment strategies.

These findings add to the growing evidence that lncRNAs are critical regulators of intestinal inflammation and hold substantial promise as predictive and diagnostic biomarkers in IBD. Although several clinical and molecular predictors of anti‐TNF response have been proposed, such as CRP, fecal calprotectin, oncostatin M, and immune cell signatures, none have achieved widespread implementation due to limited specificity or technical challenges. The lncRNAs identified here demonstrate strong predictive performance, are measurable in both tissue and blood, and could be integrated into existing diagnostic workflows with relative ease. Future studies incorporating more complex machine‐learning approaches or multiomics integration in larger, multicenter cohorts and performing mechanistic functional studies to clarify whether these lncRNAs play causal roles or function as surrogate markers of underlying biological pathways will be essential to facilitate their translation into clinical practice.

## 5. Conclusion

In conclusion, we showed that the expression of lncRNAs is altered in UC patients after anti‐TNF treatment. H19 expression was positively correlated with disease activity and inflammatory markers, such as ESR and CRP, in UC patients with UC; so, monitoring changes in the expression of H19 might be a useful indicator for the evaluation of treatment efficacy during adalimumab therapy. Furthermore, our study identified H19 and TUG1 as clinically relevant predictors of anti‐TNF therapeutic response in UC, with TUG1 in mucosal tissue and H19 in blood showing the most practical utility. These lncRNAs hold promise for refining treatment selection, reducing exposure to ineffective therapy, and advancing personalized medicine in IBD. Further validation in larger, multicenter cohorts and mechanistic functional studies will be essential to facilitate their translation into clinical practice.

## Author Contributions

**Raheleh Heydari:** investigation, methodology, software, formal analysis, visualization, writing – original draft preparation. **Mohammad Javad Tavassolifar, Romina Roshannia, and Mohammad Tayefeh Norooz:** writing – review and editing. **Mohammad Hossein Derakhshan Nazari:** methodology, software, formal analysis, visualization. **Shabnam Shahrokh:** resources. **Maryam Parvizi:** validation. **Anna Meyfour:** conceptualization, supervision, project administration, resources, writing – review and editing.

## Funding

This study was supported by the Research Institute for Gastroenterology and Liver Diseases, Shahid Beheshti University of Medical Sciences.

## Disclosure

All the authors have approved the final version of the manuscript.

## Ethics Statement

This research was approved by the Research Ethics Committees of Research Institute for Gastroenterology and Liver Diseases on 04/12/2023 (Approval Number IR.SBMU.RIGLD.REC.1402.015). All methods were carried out in accordance with the approved guidelines and regulations.

## Conflicts of Interest

The authors declare no conflicts of interest.

## Supporting Information

Additional supporting information can be found online in the Supporting Information section.

## Supporting information


**Supporting Information** Table S1. The sequences of lncRNAs primers. Table S2. Basic clinical characteristics of UC responders and nonresponders to anti‐TNF‐α treatment in the validation cohort. Table S3. The correlation between the expression levels of lncRNAs and the basic clinical characteristics of UC patients.

## Data Availability

Data are available upon request from the authors.
