# Supplementary material for: TUG1 and H19 lncRNAs Can Predict Anti‐TNF Unresponsiveness in Patients With Ulcerative Colitis: A Machine Learning–Based Approach
Source: Mediators Inflamm. 2026 Feb 28;2026:9078048. doi: 10.1155/mi/9078048 (PMC12949365; doi:10.1155/mi/9078048)
Supplement: Supplementary file 1 — Supporting Information Table S1. The sequences of lncRNAs primers. Table S2. Basic clinical characteristics of UC responders and nonresponders to anti‐TNF‐α treatment in the validation cohort. Table S3. The correlation between the expression levels of lncRNAs and the basic clinical characteristics of UC patients. [file MI-2026-9078048-s001.docx]

**TUG1 and H19 lncRNAs can predict anti-TNF unresponsiveness in patients with ulcerative colitis: A machine learning-based approach**

Raheleh Heydari^1^ | Mohammad Javad Tavassolifar^1^ | Mohammad Hossein Derakhshan Nazari^1^ | Romina Roshannia^1^ | Shabnam Shahrokh^2^ | Maryam Parvizi^3^ | Mohammad Tayefeh Norooz^4^ | Anna Meyfour^1^*

^1^Basic and Molecular Epidemiology of Gastrointestinal Disorders Research Center, Research Institute for Gastroenterology and Liver Diseases, Shahid Beheshti University of Medical Sciences, Tehran, Iran

^2^Research Center for Gastroenterology and Liver Diseases, Research Institute for Gastroenterology and Liver Diseases, Shahid Beheshti University of Medical Sciences, Tehran, Iran

^3^Department of Pathology, School of Medicine, Shahid Beheshti University of Medical Sciences, Tehran, Iran

^4^General Surgery Department, Modarres Hospital, Shahid Beheshti University of Medical Sciences, Tehran, Iran

**Correspondence:** Anna Meyfour, Research Institute for Gastroenterology and Liver Diseases, Shahid Beheshti University of Medical Sciences, Arabi Ave., Daneshjoo Blvd., Velenjak, Tehran, Iran. Postal Code: 1985717413, Tel: +98 21 22432521. Email: [a.meyfour@sbmu.ac.ir](mailto:a.meyfour@sbmu.ac.ir)

**Funding:** This study was supported by the Research Institute for Gastroenterology and Liver Diseases, Shahid Beheshti University of Medical Sciences.

**Supplementary Table S1.** The sequences of lncRNAs primers

| LncRNAs | Primer sequences |
| --- | --- |
| F-GAS5  R-GAS5 | CTTGCCTGGACCAGCTTAAT  CAAGCCGACTCTCCATACCT |
| F-TUG1  R-TUG1 | ACCGGAGGAGCCATCTTGTC  GAAAGAGCCGCCAACCGATC |
| F-CDKN2B  R-CDKN2B | CGCTCCCCTATTCCCCTTAT  GACCTCGCTTTCCTTTCTTCC |
| F-CRNDE  R-CRNDE | GTTGTCACGCAGAAGAAG  TCCTATACCTTGGCTAAACA |
| F-H19  R-H19 | GGGTCTGTTTCTTTACTT  TAGCACCATTTCTTTCAT |
| F-GAPDH  R-GAPDH | CTCATTTCCTGGTATGACAACGA  CTTCCTCTTGTGCTCTTGCT |

**Supplementary Table S2.** Basic clinical characteristics of UC responders and non-responders to anti-TNF-α treatment in the validation cohort

| Variables | UCR (N=12) UCN (N=10) | |
| --- | --- | --- |
| Gender  Female  Male | 5 (42%)  7 (58%) | 4 (40%)  6 (60%) |
| Age (year)  Family history  Smoking  Smoker  Non-smoker | 32.66 ± 11.86  -  7 (58%)  5 (42%) | 33.44 ± 11.63  -  4 (40%)  6 (60%) |
| Disease duration (month) | 64.43 ± 58.59 | 45 ± 32.44 |
| ESR (mm/hour) | 33.25 ± 22.22 | 36.16 ± 18.05 |
| CRP (mg/L) | 19.35 ± 17.81 | 34.8 ± 31.55 |
| Hb (g/dL) | 11.23 ± 2.85 | 11.55 ± 2.64 |
| WBC (10^3^/μL)  MCV (femtoliters (fL))  PLT (10^4^/μL)  Serum Iron (μg/dL)  Ferritin (ng/mL)  Vit D3 (ng/mL) | 8.34 ± 3.16  85.75 ± 5.8  38.75 ± 13.58  36.66 ± 18.51  47.72 ± 38.45  30.9 ± 14.92 | 7.7 ± 0.84  87.33 ± 6.64  34.8 ± 11.23  22.85± 17.14  53 ± 63.58  23.85 ± 11.1 |

**Supplementary Table S3.** The correlation between the expression levels of lncRNAs and the basic clinical characteristics of UC patients

| Markers | H19 | | TUG1 | |
| --- | --- | --- | --- | --- |
|  | **r** | **P-value** | **r** | **P-value** |
| ESR | 0.79 | 0.01 | 0.36 | 0.33 |
| CRP | 0.77 | 0.01 | 0.008 | 0.98 |
| Age | 0.5 | 0.96 | -0.1 | 0.76 |
| Sex | 0.57 | 0.17 | 0.21 | 0.37 |
| Disease duration | -0.11 | 0.67 | 0.33 | 0.34 |
| Hb | 0.14 | 0.61 | 0.39 | 0.25 |
| WBC | 0.2 | 0.48 | -0.08 | 0.80 |
| MCV | 0.34 | 0.45 | -0.5 | 0.66 |
| PLT | -0.34 | 0.22 | 0.33 | 0.34 |
| Serum Iron | -0.66 | 0.21 | 0.4 | 0.6 |
| Ferritin | -0.25 | 0.54 | -0.23 | 0.65 |
| Vit D3 | -0.33 | 0.38 | 0.43 | 0.29 |
| Smoking | 0.018 | 0.95 | -0.14 | 0.69 |
| Endoscopic Mayo Score | 0.70 | 0.0037 | 0.55 | 0.035 |
